# Draft Genome Sequence of a Novel *Streptomyces griseorubens* Strain, JSD-1, Active in Carbon and Nitrogen Recycling

**DOI:** 10.1128/genomeA.00650-14

**Published:** 2014-07-03

**Authors:** Haiwei Feng, Yuee Zhi, Yujing Sun, Xing Wei, Yanqing Luo, Pei Zhou

**Affiliations:** a School of Agriculture and Biology, Shanghai Jiao Tong University, Shanghai, China; b Key Laboratory of Urban Agriculture (South), Ministry of Agriculture, Shanghai Jiao Tong University, Shanghai, China; c Bor S. Luh Food Safety Research Center, Shanghai Jiao Tong University, Shanghai, China

## Abstract

*Streptomyces griseorubens* JSD-1, isolated from compost-treated soil, is able to utilize lignocellulose and nitrate as its sole carbon and nitrogen source for growth. Here, we announce the draft genome map of this actinomycete. The genes participating in lignocellulose and nitrate metabolism were picked out and identified.

## GENOME ANNOUNCEMENT

Streptomycetes are the most numerous and ubiquitous soil bacteria (1). They are crucial in the soil environment because of their broad range of metabolic processes and biotransformations. These include degradation of the insoluble remains of other organisms, such as lignocellulose and chitin, making streptomycetes central organisms in carbon recycling. In addition, streptomycetes can utilize nitrate as a nitrogen resource for growth, indicating that they also play an important role in nitrogen recycling. Here, *Streptomyces griseorubens* JSD-1 could metabolize 88% rice straw (20.0 g/liter) and 95% nitrate (5.0 g/liter) within 10 days’ cultivation. However, little is known about the genomic or genetic background related to lignocellulose and nitrate utilization of this isolate.

The actinomycete designated JSD-1 was isolated from compost-treated soil in Shanghai, China, and identified as *S. griseorubens* through 16S rRNA sequencing as well as its morphological and physiological characteristics. Then genome sequencing of JSD-1 was performed using an Illumina MiSeq platform with insert sizes of 300 bp, 360 bp, and 700 bp in paired-end as well as 3 kb and 8 kb in mate-paired libraries. Assembly of all sequence reads by applying Newbler 2.8 assembler resulted in a draft genome. Glimmer 3.0 (2) was used to predict open reading frames (ORFs) with BLASTp (3) against the Nr proteins database. The functional annotation was determined with the KEGG, COG, and Swiss-Prot databases (4–6). tRNAs, rRNAs, and other non-translated genes were predicted using tRNAscan-SE, RNAmmer, and Rfams, respectively (7–9). CRISPRFinder was used to determine CRISPR repeats (10). The signal peptide cleavages sites, transmembrane topologies, and lipoproteins were predicted by the online programs Signal 4.0, TMHMM 2.0, and LipoP 1.0, respectively (11–13).

A total of 6,432,848 reads including up to 2,209-Mb clean data were generated, representing a 263.0-fold average coverage of the whole genome. The assembled genome contained 2 scaffolds and 246 contigs. The *N*_50_ length of contigs was 53,294 bp and that of scaffolds was 7,563,100 bp. Finally, we obtained the draft genome of *S. griseorubens* with a single linear chromosome of 8,463,223 bp and an average G+C content of 72.42%. The chromosome is smaller than the ones of *S. coelicolor* A3(2) (8.7 Mbp), *S. avermitilis* MA-4680 (9.0 Mbp), or *S. griseus* IFO 13350 (8.5 Mbp) (14–16). Analysis of the genome revealed that it contained 6 rRNA operons, 66 tRNA genes, and 7,159 protein-coding sequences (CDSs). For these CDSs, 4,587 proteins could be assigned to clusters of orthologous groups (COG) families. As for the subcellular localization of the proteins, 452 proteins were identified as secreted proteins, 1,386 proteins as transmembrane proteins, and 299 proteins as transporters.

Through the analysis of RNA-Seq and quantitative real-time PCR, a variety of genes involved in the metabolism of lignocellulose and nitrate were acquired. The genes participating in lignocellulose degradation mainly included those encoding exo-1,4-β-glucanase, endo-1,4-β-glucanase, β-glucosidase, endo-1,4-β-xylanase, β-xylosidase, α-glucuronidase, α-l-arabinofuranosidase, copper oxidase, dyp-type peroxidase, catalase peroxidase, and chloroperoxidase. Meanwhile, genes for nitrate reductase, nitrite reductase, glutamine synthetase, and glutamate synthase, which are related to nitrate assimilation, were identified as well.

### Nucleotide sequence accession number.

The whole-genome shotgun project of *S. griseorubens* JSD-1 has been deposited at GenBank under the accession no. JJMG00000000.
